# Theragnostic Applications of Mammal and Plant-Derived Extracellular Vesicles: Latest Findings, Current Technologies, and Prospects

**DOI:** 10.3390/molecules27123941

**Published:** 2022-06-20

**Authors:** Nada Basheir Ali, Ahmad Faizal Abdull Razis, Der Jiun Ooi, Kim Wei Chan, Norsharina Ismail, Jhi Biau Foo

**Affiliations:** 1 Department of Food Science, Faculty of Food Science and Technology, Universiti Putra Malaysia (UPM), Serdang 43400, Selangor, Malaysia; nada44basher@gmail.com; 2 Natural Medicines and Products Research Laboratory, Institute of Bioscience, Universiti Putra Malaysia (UPM), Serdang 43400, Selangor, Malaysia; chankim@upm.edu.my (K.W.C.); norsharina@upm.edu.my (N.I.); 3 Department of Oral Biology and Biomedical Sciences, Faculty of Dentistry, MAHSA University, Bandar Saujana Putra, Jenjarom 42610, Selangor, Malaysia; 4 School of Pharmacy, Faculty of Health and Medical Sciences, Taylor’s University, Subang Jaya 47500, Selangor, Malaysia; jhiBiau.Foo@taylors.edu.my

**Keywords:** exosomes, extracellular vesicles, biomarkers, functional foods, diagnostics

## Abstract

The way cells communicate is not fully understood. However, it is well-known that extracellular vesicles (EVs) are involved. Researchers initially thought that EVs were used by cells to remove cellular waste. It is now clear that EVs function as signaling molecules released by cells to communicate with one another, carrying a cargo representing the mother cell. Furthermore, these EVs can be found in all biological fluids, making them the perfect non-invasive diagnostic tool, as their cargo causes functional changes in the cells upon receiving, unlike synthetic drug carriers. EVs last longer in circulation and instigate minor immune responses, making them the perfect drug carrier. This review sheds light on the latest development in EVs isolation, characterization and, application as therapeutic cargo, novel drug loading techniques, and diagnostic tools. We also address the advancement in plant-derived EVs, their characteristics, and applications; since plant-derived EVs only recently gained focus, we listed the latest findings. Although there is much more to learn about, EV is a wide field of research; what scientists have discovered so far is fascinating. This paper is suitable for those new to the field seeking to understand EVs and those already familiar with it but wanting to review the latest findings.

## 1. Introduction

Extracellular vesicles (Evs) were first studied and described in 1946 [1] and once were regarded as a cellular waste [2]. These EVs gained considerable attention throughout the years [3] due to the active mechanism of their release from the cells [4]. These sacs were described as extracellular vesicles [5].

A recommended definition by the International Society for Extracellular Vesicles (ISEV) advised using the term extracellular vesicles as the characteristic label for particles released naturally from the cells, distinct by a lipid bilayer, and do not replicate. The short term is EVs [6]. In order to study EVs, the process should undergo characterization to identify the transmembrane luminal protein; the characterization process should include but not be limited to zeta potential, electron microscopy, and flow cytometry [7]. EVs have been described as the novel communication method between cells, either on a long or short-range signaling requirement [8]. EVs are heterogeneous sacs of membranous vesicles released from the cells into the surrounding environment [9].

The recognized role of EVs is to carry a molecular cargo including RNA and surface markers [10] originating from the mother cell and reflect their status [11] to be delivered to the receiving cells. Organisms that range from prokaryotic cells to higher eukaryotes can produce EVs. In mammals, all kinds of cells release EVs [12]. An increasing number of studies focused their interest in plant-derived EVs as therapeutic agents since plant source materials retain less toxic effects than chemotherapeutic-based therapy [13].

This review summarised the latest updates regarding mammalian and plant source EVs in terms of classification, cargo, uptake, and function. Furthermore, we reviewed the cutting-edge isolation and characterization techniques—their role as a diagnostic and therapeutic tool, safety assessment, and evolved results.

## 2. Classification and Biogenesis of EVs

**Figure 1:**
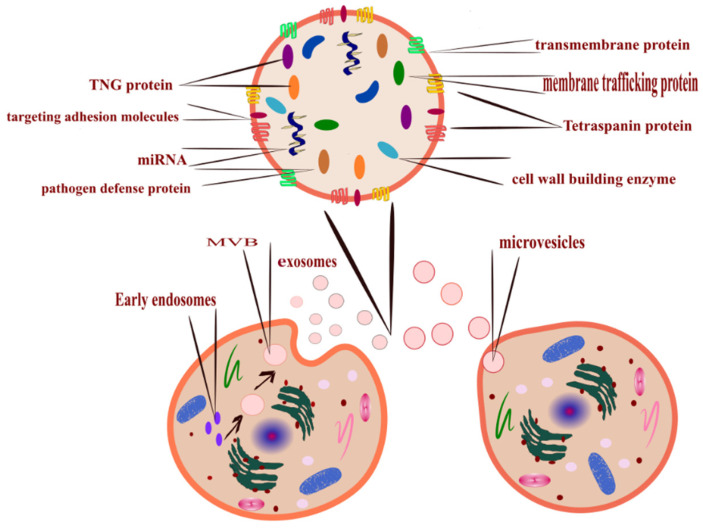
All cells release EVs, the mechanism of EVs formation determines its type, microvesicles released after budding of the plasma membrane (**right-bottom** corner), and exosomes (**left-bottom** corner) released from MVBs that formed inside the cell from early endosomes. Its cargo is loaded upon maturation (**top** corner illustrates EVs cargo). EVs can also be classified according to size; exosomes are the smallest, and apoptotic bodies are the largest.

ISEV has classified EVs based on their size into two prominent families; large EVs (200 nm) and small EVs (40–150 nm) [14]. These fluid-filled sacs can also be classified based on their biogenesis [5]. Moreover, it can be categorized and termed without complying with the standard definition [7]. The release mechanism (Figure 1) is a controlled process; lipid and lipid metabolite enzymes play the most significant role in this process [15]. There are three ways by which the cells release EVs [16].

1. Outward budding of the plasma membrane (shedding microvesicles, MVs) happens when the plasma membrane is reacting to external incitements such as inflammatory cytokines that provoke a response from the cell [17], leading to activation of the plasma membrane that will eventually release sacs 100–1000 nm in diameter that are released into the external surroundings including microparticles and microvesicles [18].
2. Inward budding of the endosomal membrane (early endosomes) results in the formation of multivesicular bodies (MVBs); these small size particles range in diameter 30–200 nm [18] and 30–150 nm [19]. The releasing mechanism of these MVBs starts by forming a sac inside the cell containing vesicles derived from the mother cell; eventually, these sacs will emerge with the plasma membrane and be released into the extracellular space, becoming exosomes [20].
3. Apoptosis-derived EVs (ApoBDs). In this case, the vesicles are large in diameter (800–5000 nm); the mechanism of release occurred during programmed cell death [21]; eventually, these vesicles will be released into the extracellular space and become another type of EV [22].

Lázaro-Ibáñez et al. [23] suggested that there is more to EVs than a sole classification into the formerly mentioned classes, stating that EVs could be allocated into further subpopulations. For instance, when the vesicles were recently examined by TEM, the EVs were described as cup-shaped particles with no indication of their cargo. Furthermore, cryo-TEM technology was used to characterize EV subpopulations. The technique confirmed the presence of cup-shaped vesicles when negatively stained; some have a single membrane smaller than 100 nm [24].

Different EV types can be produced by a single cell exhibiting diversity within the subtypes. Various subcellular locations produce subtypes of EVs, either similar or distinct [25].

### 2.1. Exosomes

Exosomes are a type of extracellular vesicles, uniform in structure and composition [26]; the diameter of these exosomes are smaller than 150nm [27]. Some studies described their size as 40–150 nm [28]. We estimate the reason behind the variation in size is the absence of standard isolation and characterization methods.

Exosomes are found in all biological fluids [29], spherical membrane-bound vesicles [30], can transfer the biological cargo into a nearby cell that is adjacent to the mother cell, or can travel long distances in the circulation [31]. The meaning of the word exosome is from the Greek words ‘Exo’ outside, and ‘Soma’ means the body [6]. In terms of structure, the exosomes’ shell is lipophilic composed of lipid membrane ligands and receptors within the membrane, RNA, proteins, and other elements derived from the mother cell [32]. The core is aqueous; its amphiphilic properties enable them to compartmentalize, solubilize and introduce hydrophilic and hydrophobic materials [33]. Exosomes represent cell biopsy, reflecting the characteristics of the original cell that can be used for diagnostic purposes [3]. The exosomal protein plays a significant role in exosome-receptor cells interaction. The protein profile of the exosomes is different in each exosome, depending on their origin [34]. The protein cargo includes but not limited to the most common CD9 [35], CD36 [36], CD81 of the tetraspanin family [37], and TSG101 [38]. As for the lipid composition, exosomes contain cholesterol, sphingomyelin, hexosylceramides, phosphatidylserine, and saturated fatty acids; all are located in the plasma membrane [39]. The cargo can be transferred between cells by a molecular sorting mechanism [40].

Exosome biogenesis is a complex process that depends on several variables, including the mother cell’s stimulating signals. They are developed and maturated from multivesicular bodies (MVs) [18]; the procedure includes a particular sorting mechanism of intraluminal vesicles [41] and segregation of the cargoes on microdomains of the membrane of the MVs, inward pudding, and fusion afterward [42]. After the formation of exosomes are the steps of miRNA loading. RNA is transported by a protein that binds explicitly with RNA. It will be carried to the lipid raft-like region for the binding process then miRNAs with the highest affinity to the lipid-like area are secured and retained [43].

### 2.2. Microvesicles

Cytoskeletal and regulatory proteins control the biological shedding of MVBs, causing a redistribution of phospholipids and contraction of cytoskeletal protein. The activities of aminophospholipid translocases cause the translocation of phosphatidylserine to the leaflet of the outer membrane forming microvesicles. Microvesicle budding, in turn, is regulated by GTP-binding protein and ADP-ribosylation factor 6. The latter is involved in cell recycling and macrophages [44]. In the last stage of MVBs release, ESCRT and TSG101 interact with ALIX and ARRDC1 (arrestin domain-containing protein 1). The lipidomic profile of microvesicles is unique. Includes phospholipid phosphatidylserine (PS) that enhances their uptake by host cells. Additionally, sphingolipids, sphingomyelins, ceramide, phospholipid lysophosphatidylcholine, acylcarnitine, and fatty acyl esters participate in their biogenesis. Furthermore, other factors affect the formation of microvesicles. For example, the concentration of calcium in the extracellular environment plays a role in the structure of MVBs. As high calcium concentration will induce the scrambling of membrane phospholipids, improving MVBs formation [45].

### 2.3. Apoptotic Bodies

Significant morphological adjustments can occur to apoptotic cells, including the blebbing and protrusion of the membrane. The process will start by shortening the cell’s nucleus, followed by blebbing of the plasma membrane, and later the cell components will be disintegrated into distinct vesicles [25]. The apoptotic volume decrease (AVD) event starts within two hours after apoptosis. The process co-occurs with the blebbing of the plasma membrane, where cysteine proteases (caspases) can be activated due to internal enticements (disfunction of the mitochondria) or directly by external factors (activation of death receptors ligations) [45]. The apoptotic bodies will be released as a result. Besides, apoptotic bodies play a role in the phagocytosis of the apoptotic cell.

## 3. EV Cargo and Uptake

Both apoptotic and healthy cells release these sacs (vesicles) containing protein, including plasma membrane and endosomal protein [46], lipids [47] most commonly cholesterol, ceramide, sphingolipids, and phosphatidylserine [48], mRNA, miRNA [49], tRNA, Y RNA [2], DNA [23], ssDNA, mtDNA, dsDNA [50] and sugars obtained from the cell f origin [10].

### 3.1. EV RNA

RNA sorting into EVs is not fully understood, but several mechanisms could achieve the process. For example, the selective loading of miRNA into EVs is facilitated by RNA-binding protein, such as SUMO protein (hnRNBA1); this protein will identify GAGAG motifs of the miRNA and selectively load them into EVs. Furthermore, the production of neutral sphingomyelinase 2 (nSMase 2) by ceramide significantly impacts loading miRNA into EVs [25]. Another example, in liver functional cells hepatocyte, the synaptotagmin-binding cytoplasmic RNA-interacting protein (SYNCRIP) is able to recognize GGCU motif in particular miRNA and augment their loading into EVs. Leading to the understanding that RNA binding proteins regulate the internalization of RNA inside EVs via miRNA-conserved motifs [51]. Once inside the recipient cell, EVs will release their cargo that will control gene expression through de novo translation and post-translational regulation of target mRNAs. Additionally, EVs can induce phenotypic changes due to their ability to change the recipient cell transcriptome and signaling activity [52].

Mammalian EVs are known to mediate the transportation of coding and non-coding RNAs. These significant components are protected inside the EVs and resume their function upon arrival to the host cell, imitating the mother cell. Still, EVs are enriched with mRNA and miRNA. Several non-coding RNAs have been detected, including transfer RNA (tRNAs), ribosomal RNA (rRNAs), and other types that are both a combination of coding and non-coding RNAs [53]. Extracellular RNA (exRNA) is usually enclosed by a membrane or found tightly associated with proteins [54].

The most abundant EV RNA is miRNA. The profile of the vesicle miRNA is different from their cell of origin. Indicating that miRNA is specific for EVs, and their sorting mechanism is selective. On the other hand, mRNA represents the minority of EV RNA. However, their concentration varies amongst EV types since mRNA is more abundant in MVBs than exosomes. EV mRNA can cause phenotypic changes in the receiving cells upon delivery. For example, the human telomerase reverse transcriptase gene (hTERT mRNA) can be localized into fibroblasts when transferred via EVs; upon entry, it enhances the cells’ multiplication life span, delays aging, and prevent the damage caused by DNA [55]. Another example is the presence of non-coding RNAs in glioblastoma-derived EVs; these EVs contain miR-21 that can trigger vascular endothelial growth factor signaling in human brain cells used as an angiogenic factor produced by the tumor cell. Furthermore, EVs derived from breast cancer cells enhance brain metastasis by changing the permeability of the brain [56]. EV mRNA can produce functional proteins; the active translation can occur within an hour after EV internalization [57].

### 3.2. Protein

The proteomic profile of EVs indicates that heat shock protein, cytoskeletal protein, transmembrane protein, and cytosolic proteins are the most abundant in EVs. Several proteins are considered markers for different types of EVs [28]. Common EV markers are listed in Table 1.

Protein is essential when EVs are being formed and released. During EV release, specific proteins are needed for membrane formation and to finish the curving and pudding of the plasma membrane; after this, protein participates in the separation process and pinching of the formed membrane. Furthermore, protein is responsible for fusing MVBs with the plasma membrane. In turn, these processes are done sequentially; they mostly require the same protein [58]. Lipids, tetraspanin, and ESCRT regulate the sorting of proteins into EVs. Other proteins packed into EVs are the results of their biogenesis process. Post-translation modifications (PTMs) similarly control the selective process of protein sorting into EVs since they regulate the protein’s function, structure, and subcellular localization [25].

### 3.3. EV Uptake

EV uptake can be divided into three primary levels: cellular, intracellular, or tissue. On a cellular level, EVs appear to interact with the receptors on the plasma membrane. The intracellular level uptake typically occurs via endocytosis, each cell with a specific endocytic pathway. The ability of EVs to transfer their RNA into receiving cells indicates that these vesicles prompt endogenous mechanisms for cargo delivery. Finally, an example of tissue-level uptake is EVs’ ability to cross the blood-brain barrier; these EVs facilitate the intracellular communication between neuronal cells [51].

The type of recipient cells appears to control how EVs are taken up. Most commonly, phagocytosis is the mechanism behind the cellular uptake of EVs. Moreover, the magnitude of the process depends on the recipient cell’s phagocytic capacities. On the other hand, the direct fusion of EVs with the plasma membrane can only occur in acidic conditions, such as the case in tumor cells [59]. When The endosomal/lysosomal system takes up EVs, Membrane-associated proteins also appear to be involved in EV uptake into cellular compartments [60]. On the other hand, endocytosis is the most recognized EV uptake mechanism; it is an active engulfment process that incorporates clathrin-mediated endocytosis, macropinocytosis, or phagocytosis. Still, it is not fully understood if the mechanism relies on specific EV-surface proteins or receptors [61].

Additionally, cell-specific factors can control EV uptake; this could be via direct contact, uptake, fusion, degradation, or a combination. For instance, protein interaction between EVs and host cells is a regulating factor in direct contact interaction. Furthermore, the lipids on the EVs membrane could be recognized by cells permitting non-specific uptake or fusion [60].

Some reports confirmed that a saturable transport mechanism mediates the uptake of EVs by the Caco-2 cell line. Furthermore, the protein contents of both EVs and the Caco-2 cell line affect EV uptake [62]. Additionally, these plant EVs have a therapeutic impact on cancer cells, such as the case in a study conducted by Zhang et al. [63] used the density gradient ultracentrifugation method to isolate ginger-derived EVs. A colon cancer cell line was treated with these vesicles to evaluate their uptake and therapeutic effect. The vesicles were taken up by cancer cells, and traces of plant-derived lipids were detected in the treated cell line, confirming these vesicles’ ability to transfer their cargo. Further investigations confirmed that these EVs have no toxic effect on human cells [63].

The last few years offered several EVs isolation methods that provided an excellent yield and promising quality isolates. Nevertheless, further analytical and comparative studies are needed to aid the scientists in determining their experimental approach [64]. Furthermore, the need for standard isolation, characterization, and storage guidelines is most urgent since the comparability between different studies cannot be achieved since the accuracy of the reported data are unknown [65].

The International Society for Extracellular Vesicles (ISEV) conducted a survey that invited the leaders in the field to answer questions related to EV biogenesis, cargo loading, release, and uptake by other cells. The answers were collected and vetted regarding the uptake mechanism of EVs.

It remains largely unclear how EVs interact with cells, and what dictates the next step (signaling, uptake, or fusion) of the bound EVs. The molecular drivers (e.g., proteins, lipids, sugars, nucleic acids) of EV-cell interactions are largely unknown. including how these vary among cell types, with cell state, or among EV subtypes [60].

## 4. Plant-Derived EVs

Human and animal EVs were the subject of research, but plant-derived EVs and their cargo are also the focus of research (Table 2). Plant extracellular vesicles demonstrated therapeutic functions via the oral route in animals. Additionally, plants are readily available, been consumed daily without any toxic effect or immune response in humans, and their assessment is the subject of many studies [70]. Plant-derived EVs were evaluated for their therapeutic activities towards tumor cells and their abilities to be used as drug carriers. These EVs were extracted from edible plants [71]. Only recently did plant-derived EVs enter the recruitment processes for clinical trials.

For example, the studied edible plants included corn, tomatoes, tobacco, sunflower, rice, and grapefruit; the latter showed an immune modulation effect in the intestine [72].

Additionally, lemons and grapes-derived EVs were isolated, characterized, and thoroughly studied to establish their beneficial effect [73]. Gut bacteria can take up plant exosomes like nanoparticles, contain RNA and siRNA in vitro, and modulate their communication with the host cells by altering their genetic makeup. These EVs, particularly their RNA, instigate tissue repair and antimicrobial immunity [74].

Moreover, Wang et al. [75] engineered grape fruit-derived nanoparticles to be delivered to tumor mice model intravenously. The nanoparticles transported chemotherapeutic agents to target cells showing enhanced efficiency and specificity. Furthermore, the safety of these nanoparticles was tested in vivo; the results showed no toxicity. Furthermore, these vesicles originated from a natural source; they provided efficient drug delivery with no toxic effect on humans [76].

EVs derived from plant sources have a structure and cargo similar to EVs isolated from mammal cells. Furthermore, the cargo of plant EVs contains proteins, bioactive lipids, and both mRNA and microRNA, and can transfer this significant cargo to other cells, just like mammal EVs [13]. One of the research subjects was corn, tomatoes, tobacco, sunflower, rice, and grapefruit; the latter showed an immune modulation effect in the intestine. There are also clinical trials using plant EVs to prevent cancer [72]. Gut bacteria can take up plant exosomes like nanoparticles, contain RNA and siRNA in vitro, and modulate their communication with the host cells by altering their genetic makeup. These EVs, particularly their RNA, instigate tissue repair and antimicrobial immunity [74].

## 5. EV Function

EVs can travel long distances through the blood circulation and reach various tissues affecting the signaling process [82]. EVs can modulate several pathological and physiological functions [83]; both actions can be used as disease biomarkers, giving them a significant advantage in the biomedical field [84], including immune function and metastasis [85]; this is achieved by transporting their cargo between cells [86] (Table 3).

The cargo within the EVs can interact with the receiving cell, causing them to function as mediators [87]. These vesicles cannot self-replicate [7]; their significance is presented in the RNA cargo [54].

On the other hand, EVs play a significant role in anti-inflammatory and antiapoptotic actions [88]. It could either work as protected vesicles that expel invading pathogen from the infected cell or as vector-transmitted virulence factors that will facilitate the infection [33]; for example, CNS-derived EVs illustrate defensive functions by releasing anti-inflammatory reactions [86]. In addition to its therapeutic functions, EVs released from infected cells are capable of acting similar to the mother cell, such as viruses [89], transferring viral RNA and other pathogenic proteins [90] that behave as barriers to prevent the immune mechanism from recognizing the virus. Thus, EVs have a vital role in the process of a viral infection, such as the case in malaria, and are involved in the communication between infected cells, viruses, and healthy cells [91]. Cancer cells can direct EVs as a messenger to transfer aggressive traits to the neighbor cells [84]; also, EVs can help the mobility of cancer cells, which is accomplished by forming lumps that will ensure persistence migration of the tumor [92], several studies concluded that tumor-derived EVs are part of tumor angiogenesis [93].

In the receptor cells, EVs can bind directly to the plasma membrane by activating the surface receptors of these cells or the endocytic membrane [50]; this action will instigate the release of intraluminal content in the cytoplasm of the recipient cells; this step is a significant activity that leads to the release of miRNA and mRNA [42].

Not only do mammals release EVs, a parasite, for example, *Heligmosomoides polygyrus*, releases EVs upon entry to the host cells that block the pro-inflammatory TNF-α and IL-6 to deliver the virulence cargo to manipulate the immune response [94]. Bacteria also release EVs essential for their survival and development; these bacterial EVs are referred to as outer membrane vesicles OMVs because they protrude from the cell’s outer membrane, 20–250 nm in size [95].

## 6. EVs as a Disease Biomarker and Diagnostic Tool

There is an increased interest in using EVs as disease biomarkers and liquid biopsy due to their availability in all biological fluids and the ability to transfer its cargo intact to the receptor cell. EVs as a disease biomarker have been the subject of several studies, especially cancer research [10]. EVs gained significant interest due to their relationship to pathological processes and gene delivery in short or long distances [109]. When EVs indicate dynamic changes in their content, they are directly related to the mother cell and their pathological condition, reflecting reliable disease biomarkers [110].

Furthermore, their non-invasive traits as disease biomarkers because of their miscellaneous content and their variety of biologically active components [15]. They consist of overlapping content, and their communication and functional mechanisms are not entirely different amongst all EV types. Providing a comprehensive investigation tool for some diseases, including cancer, cardiovascular diseases, neurological diseases, and infection [5]. Indicating that the detection of mutated EV-RNA is an effective tool to diagnose cancer [111].

Since cancer cells also produce EVs rich in a mitochondrial membrane protein that can be isolated from plasma that increases in melanoma, ovarian, and breast cancer [112]. There are specific features explicit for tumor-derived EVs that were the focal point of the focus of several studies as non-invasive diagnostic tools. These markers include epithelial cell adhesion molecules, epidermal growth factor receptors, and mucin [113]. EV-enriched proteins play a role in cancer development and can be used to diagnose metastatic cancer with a weak prognosis [114]. Tumor EVs are detected in almost all bodily fluids, including blood, urine, and cerebrospinal fluid. Confirms that EVs are circulating cancer biomarkers, as they represent in liquid biopsy in the breast, prostate, ovarian cancer, and melanoma [39].

The detection of EVs and their subset can predict diseases and anticipate the risk factors or development of a pathological process. Hence, EVs have also been used in a Framingham risk score (FRS) tool, a risk assessment approach [115].

The appropriate approach to use EVs in diagnosis is to study the nature of these vesicles and their origin. For example, analysis of plasma EVs confirmed their involvement with a specific type of cancer. Furthermore, some biomarkers are released directly from the cancer cell; others can be identified by their specific protein. All these indicators and tools can be incorporated with traditional tools to study cancer prognosis, development and, possible prediction [116].

For example, EVs were tested as early non-invasive diagnoses of colorectal cancer (CRC); the isolated EVs expressed tumor-specific protein circulating extracellularly, which can be utilized as early stages diagnostic tools [117]. Other than employing EVs in cancer diagnosis, some research focused on using EVs to diagnose rapidly progressed diseases that instigate a challenge for early diagnosis. Such is the case in Pleural effusion; EVs can be a promising source to detect this disease. After metabolic and lipidomic characterization of Pleural effusion-derived EVs, the results indicated that these EVs are enriched in specific metabolic cargo with a noticeable variation from tuberculosis-derived EVs and malignant tissue, indicating a promising future to use these particular characteristics as an early diagnostic tool [118].

In a study conducted by Saenz-Pipaon et al. [10] aiming to evaluate the potential use of EVs as a diagnostic tool for Peripheral Arterial Diseases PAD associated with cardiovascular conditions, the study concluded that EVs are likely reliable liquid biopsy to predict the PAD molecular component signature. Additionally, EVs play a significant role in the lungs by controlling the airways’ homeostasis and providing a significant circulating disease biomarker in chronic obstructive pulmonary disease and potential therapeutic due to its cellular communication abilities [38].

Cheng et al. [119] conducted detailed profiling of EVs collected from the frontal lope of Alzheimer’s patients. The study investigated the RNA makeup of these EVs and the changes associated with disease prognosis. The results indicated that EVs are involved in Transcriptomic deregulation of miRNA expression, achieved by horizontal transfer of the RNA via EVs. The study suggested that these early changes can be used to indicate and diagnose Alzheimer’s [119]. EVs also helps in providing long-term disease monitoring and predicting possible relapse. Moreover. A study by Melo et al. [120] acknowledged that the number of exosomes was significantly elevated in vivo in pancreatic cancer before the disease was detected by screening techniques [120].

## 7. Application of EVs as a Therapeutic or Drug Delivery Agent

**Figure 2:**
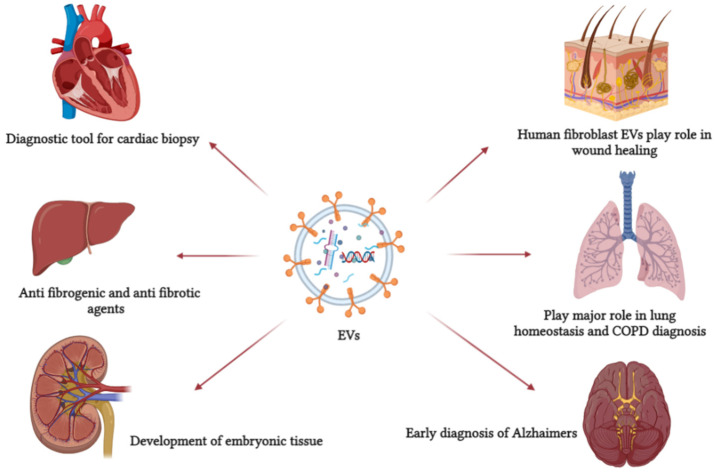
EVs are involved in several pathological and physiological processes. It can be used as a primary non-invasive diagnostic tool and, because of its role in cell communication, it can be used as a drug carrier. This figure refers to some EV functions in defense mechanisms, cellular communication, and normal physiological processes.

Evidence showed that EVs function systematically as a natural response and drug delivery vehicle and can be employed for target delivery (Figure 2). EVs are detectable in most biological fluids in disease and immune response [121]. EVs can be used as original therapeutic agents or as delivery agents. Most studies focused on the possibility of using them as drug delivery cargo to a specific target due to its membrane protein [67]. Once the drug is encapsulated inside EVs, the advantage is that the drug works with the elements naturally present in them. In other cases, EVs only act as a drug carrier protecting the drug, passing it safely to the target location [73].

Several studies indicated that EVs derived from stem cells could be used as a non-invasive treatment for a brain injury or brain infection, as these EVs retain the same therapeutic functions as stem cells. Furthermore, the secretomes of stem cells, including EVs, are the active compounds that attribute to their therapeutic roles; in addition, stem cell-derived

EVs can transfer the required genetic information and protein to accomplish the therapeutic process. Several studies applied stem cell-derived EVs on injured brain cells; the EVs acted as therapeutic agents on these cells [122]. Additionally, immune cells also release EVs that act similarly to their origin and can be used in therapeutic applications. For example, dendritic cells (DC) release EVs that activate CD4+ T cells [67] via the endocrine pathway, causing enhanced cardiac performance and reducing the time needed for wound healing in myocardial infarction. EVs derived from macrophages are used in plasmin encoding therapeutic protein and vaccination [123]. Salivary EVs prevent the Zika virus attachment to the host cell, explaining why the virus is rarely transferred via saliva. However, the same study stated that the salivary EVs are not effective against SARS-CoV-2 that majorly communicate via salivary droplets [124].

There are a variety of challenges facing scientists when developing a therapeutic approach. These included toxicity, safety, target specificity, and large-scale production. Furthermore, a synthesized nanoparticle can solve the target delivery issue. However, it must undergo in vivo safety and toxicity assessment, and there is the issue with costly large-scale production. Using extracellular derived nanoparticles as drug carriers provides a possible solution since they contain lipid, RNA, and protein [63]. As EVs transfer their content from the mother cell, it is the most critical attribute in drug delivery and cell communication [36]. Their cargo, specifically RNA, was of particular interest due to the diverse population of miRNA with target genes that can influence and regulate the biological process in mammalian cells [125]. Additionally, a growing body of facts indicates that cell apoptosis proteins are involved in EV formation. Unveiling the interaction pathway between EVs and autophagy that promotes cancer cell motility, providing significant advantages in early diagnosis and cancer treatment using EVs [126]. The protein profile of EVs derived from mesenchymal cells retains a therapeutic effect observed in vivo. These proteins include osteoprotegerin and angiogenin [127].

Drug loading into EVs can be divided into two strategies. The first is directly loading the drug into the exosomes; the second is targeting the mother cell by loading drugs during the biogenesis of exosomes [34]. Furthermore, in the case of loading lipophilic drugs, the process is relatively easy due to the interaction between EVs lipid bilayer and the drug, which occurs via hydrophobic interaction [128]. Several methods were utilized to achieve exogenous loading of EVs with drugs, including electroporation, incubation, sonication, and thawing. The level of success differs from one method to another; some cause degradation to either EVs or their cargo [129].

The first time EVs were used as a drug cargo was when San et al. [130] used EV-encapsulated curcumin fused with the plasma membrane. It was reported that EVs isolated from the plasma of humans and rats showed a protective mechanism against Acute myocardial ischemia/reperfusion [131]. Successfully, EVs were loaded with anti-cancer and RNA-based drugs and cancer vaccination. These applications are based on cancer immunotherapy, indicating that the success of this approach is that EVs can be taken up efficiently by macrophages and tumor cells [132]. Other drugs loaded into EVs include a lipophilic drug with a small molecular weight and RNA-based drugs, for example, small interfering RNA (siRNA) [128]. Furthermore, the blood-brain barrier is the most selective in the human body. There are three primary methods to deliver drugs and therapeutic components into the brain, including invasive, pharmacological, and physiological approaches—the first approach is based on delivering drugs by breaching the blood-brain barrier. The pharmacological method includes modifying the active compound to enter the brain via a passive crossing. The last approach is the most efficient; it depends on the naturally present receptors found in abundance on the surface of the blood–brain barrier. These receptors provide easy and equal distribution of the loaded drugs into the brain. EVs possess two significant advantages as the usage will be non-invasive and were proven to cross the blood-brain barrier easily [133].

Employing EVs as a drug carrier has several advantages compared to platelets. To mention a few, (1) EVs are smaller in size, which is an advantage when delivering drugs to cancer cells. For instance, EVs were more efficient in delivering the membrane protein SIRPa than ferritin. (2) EVs are originated from different sources. They are smaller and less toxic than lysosomes, with enhanced tissue biocompatibility and tolerance. (3) The cargo within EVs is protected by the double lipid layer that reduces the degradation and improves their biological stability. (4) Target cells can exclusively distinguish the specific receptors present on the EV membrane, reducing systemic toxicity and lowering drug outflow compared to conventional drug administration methods. (5) It is established that EVs could pass the BBB, giving an advantage in treating brain tumors. (6) Due to EVs’ ability to circulate the blood, the drug’s effect can last longer in the system [71].

## 8. Drug Loading into EVs

Loading EVs with foreign substances for therapy continues to grow and develop [134]., enhancing the therapeutic potentials of these particles. The loading approach can be categorized into EV alteration or cellular modification [44]. Furthermore, it can be achieved post EV isolation (exogenous). Or during the biogenesis of EVs within the mother cell (endogenous) [51].

EVs were tested to be used as a vehicle for drug delivery systems. One of these applications was developed by delivering systemically injected exosomes to target epidermal growth factor receptors in breast cancer; the study stated that these exosomes successfully conveyed miRNA and anti-tumor agents into epidermal growth factor receptors [135]. Conjugating gold nanoparticles developed another system into the doxorubicin anti-cancer drugs that were physically loaded into the exosomes. The study stated that the target cell successfully took up these modified exosomes [136]. Another modified EV consists of exosomes-liposomes fusion by repeated freeze-thawing mechanism; this method successfully altered the treated exosomes surface, resulting in enhanced colloidal stability and reduced immunogenicity [135]. The significant advantage in using EVs as a drug delivery agent or as a therapeutic agent on their own is the ability of these EVs to control their RNA, proteins, and lipids and employ it as a tissue-specific response [121]. Furthermore, EVs are being engineered and modified to enhance the encapsulation of the drug and the targeting accuracy [137].

Although EVs proved to have significant targeting abilities, some studies indicated that EVs were accumulated in the spleen and liver and later removed by macrophages. Two techniques were developed to correct the off-target internalization of EVs. The first is based on modifying the surface of EVs using polymers, increasing the duration of EV blood circulation and possibilities of target accumulation. The second approach is based on modifying the surface of EVs by adding a part of a molecule that has an affinity to the target cell or tissue [138].

### Advantages of EV-Drug Delivery Technique

The internal cargo of EVs is protected by their membrane, likely by encapsulation, primarily originating from the cytosol of the mother cell, which will be transferred to the recipient cell upon fusion. Since the intraluminal vesicles are originated in the acidic condition of the multivesicular body, EVs and their protein should be resistant to pH. Additionally, upon entering the endosomal pathway, EVs will also undergo progressive acidification [139].

In addition, since EVs are encapsulated phages, some demonstrate higher tolerance for harsh conditions, providing higher efficacy when orally administered. The pH conditions in tumor cells may range from 6.0 to 6.8. Initial data concluded that cancer cells cultured in a physiological pH 7.4 released a significantly lower concentration of EVs than those cultured in 6.4 pH. Concluding that, cells release EVs at low pH to avoid toxin accumulation [140].

Ogawa et al. [139] studied the stability of EVs under different pH conditions. The study stated that EVs are stable in functionality and shape under acidic conditions for at least 3 h. In case of EVs rupture, the action can be due to membrane penetration by protease. The process is facilitated by fatty acid cleavage mediated by phospholipase A2, leading to EV surface protein degradation [139]. The surface protein profile on the EVs’ membrane is important due to proteases damaging the extracellular environment. For example, the IgA present on the surface of EVs protects these vesicles from breaking down [141].

## 9. EV Isolation Techniques

Several methods to isolate EVs are available. Such methods are ultracentrifugation isolation and microfluidic-based isolation [142], polymer-based precipitation, ultrafiltration, and immunoaffinity column, which can isolate EVs based on their size. All of which supplies plusses and minuses [106].

A study conducted by Buschmann et al. [64] aimed to compare different EVs isolation methods and their miRNA in terms of yield and quality. The isolation protocols used in this study included ultracentrifugation, precipitation, sedimentation, and size exclusion chromatography, along with a commercially available isolation kit. The study concluded that each isolation method provided a method-specific difference, including isolates size, yield, and purity of miRNA, indicating that each method can isolate a particular population of EVs with specific contaminants.

Antibody-based isolation methods employ a chosen biomarker present on particular cells’ released vesicles’ surfaces. This method has its drawbacks, including the inability to isolate vesicles that carry more than one protein biomarker on their surface [143].

### 9.1. Centrifugation Method

**Figure 3:**
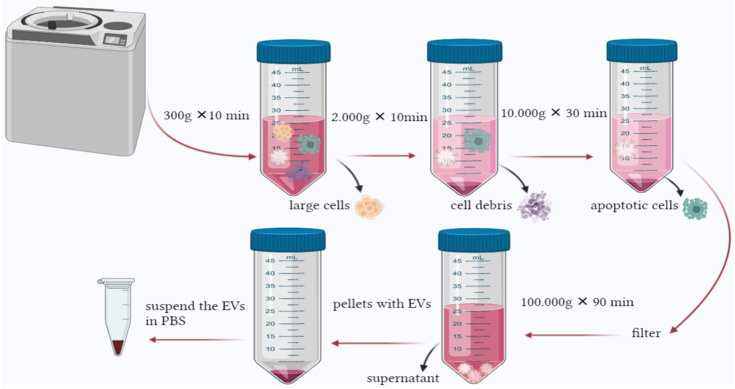
Ultracentrifugation is the most common method used to isolate EVs. The process is based on increasing the force and time gradually to pellet large cells and contaminants until reaching the final centrifugation, where EVs can be found in the pellet.

This method is declared to be the golden protocol to isolate EVs. However, its time consuming and depends mainly on the instruments needed. A fast and practical approach is required to facilitate EVs’ isolation [142]. Several studies used similar centrifugation speeds ranging between 1500× *g* and 125,000× *g* [144]. In general, the method isolates EVs based on their density. The speed gradually increases to sediment the large cells, cell debris, and apoptotic bodies. The process incorporates several steps starting with a speed as low as 300× *g* for a short period to pellet the cells, then 2000–10,000× *g* to remove apoptotic bodies and vesicles larger than EVs. The obtained supernatant will be ultracentrifuge at 100,000–200,000× *g* in order to pellet EVs. The obtained particles should be subjected to microfiltration to remove impurities [145] (Figure 3). Protein profiling of EVs isolated from urine by two methods was performed by Bijnsdorp et al., [146] where ultracentrifugation and heat chock proteins Vn 96-peptides were performed separately. The proteomic profiling was undertaken by mass-spectrometry analysis. The results indicated that protein profiling of EVs from both methods was similar; also, the Vn96-peptide methods are less time-consuming and can yield higher EVs.

#### Optimization of Ultracentrifugation Isolation Method

A common modification to the ultracentrifugation method is to combine it with a sucrose gradient. The sucrose gradient is the purification step. This method allows the vesicles to float into a laid sucrose gradient. EVs markers can be observed and trapped within the different gradient layers, as reported in many studies [147]; this method was applied by Silverman et al. [148] to isolate and purify EVs derived from frozen neural tissue from both human and murine.

Somiya et al. [101] developed an approach to improve EVs’ yield and purity isolated by ultracentrifugation; the extra step included treating the yield by acid to remove impurities and non-EV protein followed by additional ultracentrifugation.

Before conducting a differential centrifugation process, another treatment was experimented with by treating the tumor sample with enzymes (collagenase D and Dnase) to isolate a pure EVs subpopulation with intact characteristics. Electron microscopy analysis indicated that the isolated EVs are intact, pure, and maintained the morphological characteristics of the vesicles and that enzyme treatment enhanced the isolation of EVs from dense fibrotic tissue [18].

A combination of ultracentrifugation with subsequent agarose gel electrophoresis to isolate dry plant EVs was developed by Woith et al. [149]. The approach included a series of centrifugations, the protein contaminants were eliminated, and the final isolates obtained from 5000× *g* pellet were applied to agarose gel to remove the soluble contaminants. In order to recover the pure EVs, the gel is sectioned and removed by an additional final centrifugation step. Results indicated that impurities passed through the gel while EVs were retained in the gel pockets due to their smaller size.

Ultracentrifugation methods were compared with Optiprep™ Density Gradient ultracentrifugation in terms of purity and quality of the isolated EVs. The study isolated CNS-derived EVs from microglia, isolated EVs from both methods were characterized using mass spectrometer analysis indicated that Optiprep™ Density Gradient ultracentrifugation provided richer isolates with a limited amount of co-protein contaminants, but the TEM analysis detected aggregated vesicles within the isolates [150].

### 9.2. Size Exclusion Chromatography Method (SEC)

The method is well-established that separates macromolecules based on their size and volume. Typically, the system includes a stationary phase containing cross-linkage agarose beads commonly used for EV isolation [151].

A study combined size exclusion with ion-exchange chromatography methods to investigate miRNA and protein quality and assess the isolated EVs’ biological activities. The study concluded that the isolated EVs were rich in miRNA and proteins and were biologically active [122]. Bind-elute size exclusion chromatography (BE-SEC) combined with tangential flow filtration (TFF) is an optimized protocol to purify the isolated EVs from residual soluble protein [124].

A study conducted by Takov et al. [152] aimed to evaluate the difference between EV isolation using UC and SEC methods in terms of yield, purity, and quality. NTA used for size and concentration determination, as for the protein count and concentration BCA protein assay kit was used along with proteome profiler human angiogenesis essay, along with other characterization and profiling approaches to conclude that the EVs isolated from ultracentrifugation are less in terms of yields. However, the quality and purity of SEC isolated EVs are questionable.

### 9.3. Immuno-Affinity Based Column Method

The immuno-affinity based column method is employed to capture EVs based on particular surface markers; the method was reported to isolate pure EVs successfully. However, a significant obstacle is the lack of plant EV particular markers [121]. Still, the affinity-based method is an excellent tool for understanding the function of EVs. A study conducted by Nakai et al. [35] aimed to develop a new affinity-based EV isolation method from biological fluids and conditioned media. The study used T-cells immunoglobulin and mucin containing Tim4 protein; the isolated EVs were reported to be pure, intact, and easily separated from the capturing protein by adding buffer with a chelating agent.

Affinity-based aqueous two-phase systems (ATPS), albeit not commonly used, was described as an efficient and fast isolation method with fewer contaminants, used to isolate small EVs from plants, parasites, and cell culture. It combines two polymers with salt solutions that separate and isolate various biomolecules optimized to remove protein contaminants. The most significant advantage when using this method is that shorter time utilized with pure and intact yield; also, there is no need for specific equipment to use ATPS. The primary disadvantage is the presence of dextran in the final suspension of EVs [153].

## 10. Characterization of EVs

The size of EVs in general and exosomes, in particular, is so small, making accurate characterization of EVs very challenging. For instance, nanoparticle tracking analysis will not detect particles smaller than 50 nm; on the other hand, flow cytometry will only detect particles larger than 300 nm. Dynamic light scattering will detect particles varied in size between 1 and 6000 nm with overestimating the particle count. Another option is using atomic force microscopy and electron microscopy. However, these methods are time-consuming, costly, and require professionals to operate the device. For example, if the sample concentration is high, the support film will be damaged, preventing EV counting and imaging since the characterization methods are not standardized and integrated. The data available in the literature are diverse and conflicting [71].

Physicochemical and biochemical characterization of EVs aims to identify the isolated population and elucidate their profile in terms of lipidomics, proteomics, and gene expression analysis [154]. Physicochemical characterization of EVs studies their size [155], morphology [156], cargo, or the number of vesicles; the latter can be achieved by zeta potential analysis [157]. Size characterization is commonly achieved using dynamic light scattering techniques [158], light microscopy, and electron microscopy [21]. Vesicle concentration and size distribution are studied using nanoparticle tracking analysis [159,160] and flow cytometry [105].

Cry electron microscopy characterizes the particles without adding heavy metals to the sample as fixative agents. This method was used in a study to characterize single cell EVs in terms of their morphology, structure, and lipid layer. The cry electron microscopy provided information confirming that single-cell EVs showed diverse characteristics [26].

Flow cytometry has advantages and disadvantages. The functioning concept is detecting the vesicles’ fluorescence and light scattering signal individually, but the technique does not detect all EV subclasses. Therefore, the quality of the results depends on the device’s sensitivity since EVs concentration increases when their size decreases [161].

Additionally, proteomic characterization is a practical approach to detect common EV markers. The most common proteomic characterization is conducted by mass spectrometry [101]. Label-free quantitative proteomics is a mass spectrometry approach that detects common EV protein markers regardless of their morphology, origin, or cellular strain. This method can detect plasma membrane protein, tetraspanin, and GTPase [162]. Liquid chromatography-tandem mass spectrometer (LC-MS/MS) analysis also characterizes EV protein concentration and cargo [150].

Characterization of EVs based on their genetic makeup can be achieved by running a quantitative real-time PCR; the analysis takes place after extracting and amplifying the RNA to detect the genes present and their respective metabolic process [105]. Ex vivo behavior and communication of EVs, especially tumor-derived EVs, are investigated by applying 2D and 3D cell culture models; these two techniques are significant for evaluating RNA-derived EVs. Although, the 3D cell culture model is more competent when evaluating the shape of the vesicles, their behavior, and their extracellular communication [49].

## 11. Toxicity and Safety Assessment of EVs

Due to the endogenous properties of EVs, they migrate between cells and deliver their biological cargo upon crossing the membrane [86]. EVs can travel long distances through the blood circulation and reach various tissues affecting the signaling process [82], mediating several pathological and physiological functions [83]. On the other hand, EVs play a significant role in anti-inflammatory and antiapoptotic actions [88]. It could either work as protected vesicles that expel invading pathogens from the infected cell or as vector-transmitted virulence factors facilitating the infection [163]. Meaning that the function of EVs upon entering the body depends on the biological status of the host and the receiving cell. There are cases when EVs act as immune responses against infection. Furthermore, it worked as a signaling pathway for pathogens [164].

Studying the efficacy of EVs in therapy is significant, especially in preclinical development. However, evaluating their risk and toxicity is essential in developing their therapeutic performance. Attention must be directed towards the possibility of immunogenicity or toxicity of therapeutic EVs [165]. Therefore, an in-depth understanding of EVs’ overall toxicity and biodiversity following in vivo studies is required before using them as therapeutic agents or drug carriers, primarily if the EVs are originated from xenogeneic sources [166].

Somiya et al. [101] evaluated the safety of milk-derived EVs; the paper stated that no systematic toxicity or immunogenicity was detected. The mice were given an intravenous injection containing EVs derived from milk. A blood sample analysis confirmed the safety of these EVs, and no toxicity markers from the kidney nor the liver were observed.

A study conducted in vitro evaluation of the toxicity of mesenchymal and bovine milk-derived EVs stated that there is no genotoxic response to either EVs, but collagen-induced platelet aggregation in a dose-related manner was recorded. In vivo investigation was conducted to assess the safety of engineered HEK293T cell-derived EVs via intravenous administration for 21 days. The results provided no signs of toxic effect, immune changes, and no alteration of the EVs [167].

According to a study conducted by Roccaro et al. [168]. EVs were isolated from Mesenchymal stem cells obtained from multiple melanoma patients. The results indicated that EVs promote tumor growth by dissemination and metastasis to distant multiple melanoma niches. Besides, EVs are associated with stimulating or suppressing the immune response and play a role in regulating the suitable microenvironment for the tumor’s biological process [163].

Plant-derived EVs pose several advantages over mammalian-derived EVs. Therefore, the field experts recommended an accurate assessment of their toxicity and safety for clinical application [169]. Engineering EVs into a therapeutic tool is valuable, ensuring quality and safety. in the time being, most available data on the safety and toxicity of EVs in vitro and in vivo are using mesenchymal stromal cells-derived EVs. Not enough attention was dedicated to other EVs from different sources [166].

**Figure 4:**
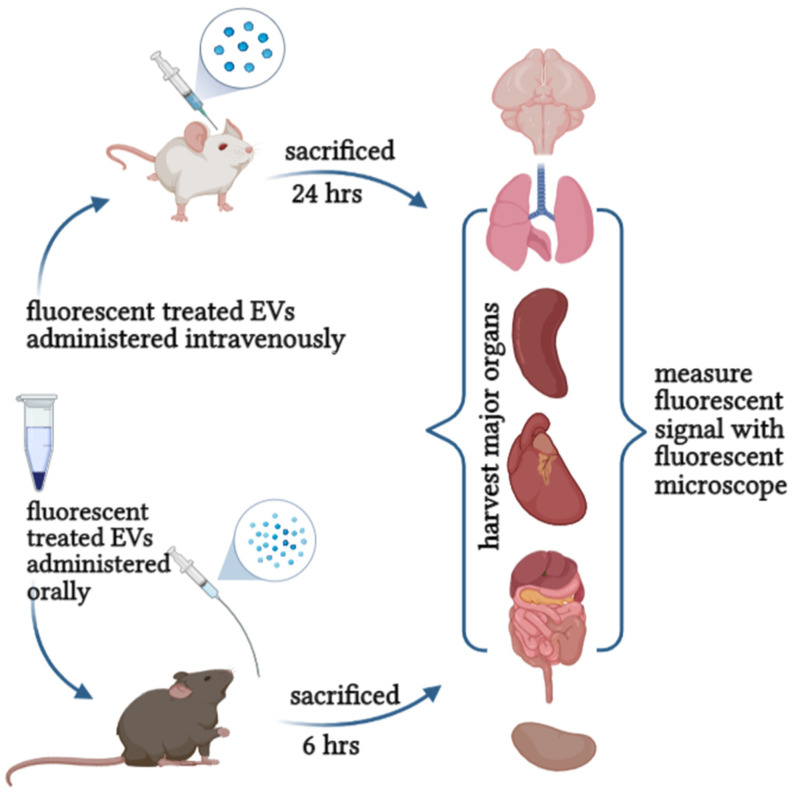
Evaluation the safety and toxicity of EVs can be achieved either orally or intravenously.

Therefore, evaluating the toxicity and safety of EVs in vitro and in vivo will set the dose for future clinical use and safe consumption recommendations for human application. It provides fundamental insight into pharmacodynamics, therapeutic benefits, purity, ideal storage, shelf-life, and quality control (Figure 4). These quality and safety control processes will innovate the possibilities of applying plant-derived EVs in therapy and establish regulations and standards for production and development.

Although, there are no set standards or parameters to evaluate the safety and toxicity of EVs. However, understanding their uptake and behavior locally and systematically is vital to assess their safety [170].

## 12. Storage Recommendations and Stability of EVs

EVs’ storage conditions significantly impact their therapeutic value and stability [171]. Many studies showed uncertainty regarding the storage stability of EVs under different conditions, particularly prolonged storage. Mainly focusing on the degradation of protein using western plot analysis but failed to focus on their functional ability after its uptake by other cells. Sterzenbach et al. [172] tested the biological function of EVs after storage; they indicated that EVs showed a degree of sensitivity and fragility compared to newly isolated ones; there was also a reduction of EV ability to transfer their protein to the receptor cell [172]. Rutering et al. [173] isolated EVs from bovine milk and stored them for 18 months at −80 °C, the study claimed that this storage condition had little effect on the particle size, and no coagulation was observed. Additionally, the impact of storage on the biological activity of EVs was assessed by anti-proliferation activity. The results indicated no loss of activity due to prolonged storage. The recommended storing condition of EVs as was published by ISEV is that EVs should be suspended in phosphate buffer saline and stored at −80 °C because a greater temperature causes a reduction of the number of isolated EVs [174].

## 13. Discussion and Conclusions

This review provides a concise retelling of the history, concept, development, latest findings, and recommendations related to extracellular vesicles. We recommended this paper to those new to the field needing to understand EVs and those already familiar with it but wanting to keep up with the latest findings. We listed the continuous change in technical developments, new findings, and debates along the review lines.

Starting from the early history of these riveting nanoparticles, we observed that it is well established that contrary to the previous common concept that classifies EVs as a mere cellular waste carrier, the field’s advancement unveiled that these nano-sized vesicles play an essential role in several processes [175]. One of the most is their involvement in cellular communication, transportation of miRNA, protein, lipids [60], and as a significant player in several pathological processes, including infection and metastasis [176], agreeing that EVs are an appealing tool for non-invasive rapid diagnosis that can be exploited in the case of asymptomatic diseases [177]. Another vital fact is that EVs play an essential role in embryonic growth, development, and maturation. Such as in breast milk-derived EVs, which transports EVs from the mother to the newborn [178]—confirming their contribution to pathological or physiological processes.

The reported number of searches on EVs is elevated yearly, providing more knowledge and clues to understand these nanoparticles better.

This review focused on the most recent advancement in extracellular vesicles, their function, the latest update on EVs cargo, and their contribution and involvement in physiological and pathological processes [179]. We highlighted some of the technical challenges, current findings and, breakthrough developments in research related to EVs isolation, characterization, and drug loading technology. That has been addressed by many papers [180], giving hope for more findings and better results.

A fact affirms an increased count of the number of EVs circulating in the system during infection, providing early diagnostic tools significant in cancer or sudden brain ailment [83]. Several researchers concluded that cancer cells release EVs and therefore are found circulating in the body carrying their genetic code [181]; these EVs act as a messenger, communicating with nearby or distant cells [182] during metastasis, making the surrounding environment ideal for the pre-metastatic niche [183].

ISEV visited the topic in a position paper, stated that EVs had already been used in human trials to treat cancer in early 2000 [177]. Furthermore, the available results point to the importance of the role played by plant-derived EVs in cancer studies as therapeutic and drug carriers [71]. The cargo of plant EVs is substantial and similar to mammal EVs, not to mention their use in therapy is meaningful to those who are strictly vegan or religious individuals. All are essential contributions to the fight against cancer.

Some studies used plant-derived EVs to understand their function and potential use as a drug carrier [184]. Plants EVs also act similarly to mammal EVs, albeit with a slight difference in the biogenesis, circulate, allocate and carry specific cargo [185]. The significance of plant EVs is underestimated since plants are readily available from biological origins [186]. Not to mention that the toxicity of plant source materials is not substantial, with no considerable reported side effects [187]. As a result of this review, mammal and plant EVs are similar in function, characteristics, and cargo, and can act in physiological and pathological conditions. Using these EVs in future research can majorly contribute to the field, especially if the safety of plant EVs were to be confirmed. The already-existing body of evidence on the use of plant-derived EVs for nano-delivery offers a wide range of possibilities for their future use in health and therapeutics.

Since there are several obstacles, the researchers face this field [188]. We highlighted the latest advancements, recommendations, and contributions from ISEV, the leaders in this field. ISEV stated in an early published position paper that EVs’ field faces continuous challenges technically, including the expanding number of EVs isolation methods with low yield [189]. Advising that, it is essential to accurately state whether the studied EVs are used for clinical application as the active drug component, or it is simply the drug carriers following ISEV guidelines [177].

Additionally, based on the recommendations from ISEV that were published on their position paper titled ‘Applying extracellular vesicles-based therapeutics in clinical trials an ISEV position paper’. The paper mentioned that using EVs in therapy must comply with the established regulations. Clinical investigation and quality control are crucial aspects of EV production in therapy. It is essential to accurately state whether the used EVs are the active drug component or used as a drug carrier. Their statement was:

EV-based therapeutics can be defined as biological medicine and belong to the pharmaceutical class of biologicals. Regulatory frameworks for manufacturing and clinical trials exist in Europe, Australia, and United States, but special guidelines targeting EV-based therapeutics may be needed.

However, more extensive studies are needed to fully understand the former as an active component in therapy; we recommend that added attention be directed towards plant-derived EVs, their function, cargo, and characteristics that were the subjects of a shy number of articles compared to mammal EVs. More safety assessment studies and quality control standards are additional clinical application recommendations. We observed that the lack of standard isolation, characterization protocols, and quality control hinders these studies’ advancement in either purity or yield of EVs for clinical use [190], making it hard to link and compare the available findings. Then again, the field recognized these issues [65] and they have been addressed on several occasions, emphasizing the need to standardize the isolation protocols. Although there is so much more to learn about EVs, what scientists have discovered so far is fascinating and promising. Nevertheless, this preliminary data offers additional insights into other possible benefits of EVs for a better future.

## Figures and Tables

**Table 1:** Common EV protein cargo is often used as EV markers.

Common Markers Used for the Characterisation of EVs	Reference
Cytosolic ESCRT ^1^	[66]
Rabs ^2^	
HSPA5 ^3^, cell growth proteins (GSN ^4^ and FSCN1 ^5^) ATPase family ^6^ (ATP1A1, ATP1B1, and ATP2B1) signal protein (ROCK2 ^7^, ANXA1 ^8^, CORO1B ^9^, and ARF1 ^10^)Cell communication proteins (ANXA6 ^11^, LYN ^12^, OXTR ^13^, STX4 ^14^, and GNB1 ^15^) Cation transporters	[14]
Cytoskeletal proteins (ACTG1 ^16^, DSTN ^17^, FLNA ^18^, COTL1 ^19^, KRT1 ^20^, KRT9, KRT10, MSN ^21^, PFN1 ^22^, and WDR1 ^23^)	[46]
TSG101 ^24^ and LAMP1 ^25^	[41]
Lipid raft-associated protein (Flot1) ^26^	[47]
Lipid raft cellular prion protein (PrP) ^27^	[2]
Tetraspanin	[67]
ALIX ^28^ part of EVs release	[68]
VPS4B ^29^ and HSP70 ^30^	[69]

^1^ Endosomal sorting complex required for transport, ^2^ Rab-associated binding, ^3^ Heat shock protein 5, ^4^ Gelsoline_,_^5^ Fascin actin-bundling protein 1_,_^6^ Adenosine triphosphatase protein, ^7^ Rho-associated kinase, ^8^ Annexin A1, ^9^ coronin actin protein, ^10^ ADP-ribosylation factor 1, ^11^ Annexin A6, ^12^ Lck/Yes novel tyrosine kinase, ^13^ oxytocin receptor, ^14^ Syntaxin 4, ^15^ Guanine nucleotide-binding protein, ^16^ Actin gamma 1, ^17^ Destrin, ^18^ Filamin A, ^19^ Coactosin-like protein 1, ^20^ Keratin 1,9 and 10, ^21^ Moesin, ^22^ Profilin 1, ^23^ DW repeat domain 1, ^24^ Tumor susceptibility gene 101, ^25^ Lysosomal-associated membrane protein 1, ^26^ Flotillin-1, ^27^ Prion protein, ^28^ ALG-2-interacting protein X, ^29^ Vacuolar protein sorting 4, ^30^ Heat shock protein70.

**Table 2:** Assortment of isolated plant-derived EVs and their characterization compared to mammal-derived EVs.

Plant Source	Findings	References
Ginger and carrots	The isolated EVs are similar in structure to mammal derived EVs and involved in cellular communication and plant defense response.	[63]
Leaves of Dendropanax morbifera	Similar in size to mammal EVs. Reduces the synthesis of UV-induced melanin, therefore can be used as a natural ingredient in cosmetic products.	[13]
Arabidopsis	The EVs are saturated with a protein involved in stress and immune response in infected plant.	[77]
Watermelon	The isolated EVs are comparable to animal EVs, and the predicted function includes regulation of the development, ripening, and metabolism process of the fruit.	[78]
Curcumin	Delivery agent for a cancer drug induces a low immune response and cross mammalian parries.	[79]
Cotton	Anti-fungal activity.	[80]
Coconut, kiwi, and Hami melon	Detection of over 400 miRNA and the isolated EVs control inflammatory expression and gene-related cancer.	[81]

**Table 3:** Mammal-derived EVs were thoroughly investigated; their involvement in several pathological and physiological functions was recorded in various studies.

Mammal Source	Findings	References
Brain cells EVs	Play a physiological and pathological role in the CNS.	[96]
Human breast milk EVs	Contribute to the maturation process of the newborn, intestinal development, and microbial programming of infant tissue. Modify infant immune system. Support the cellular function and growth of the infant.	[97,98,99,100]
Bovine breast milk-derived EVs	Transport RNA to the receptor cell. Immunoregulatory function.	[101]
Embryonic stem cells EVs	Enhanced proliferation of human tissue.	[102]
Pancreatic EVs	Stimulation of T and B cells.	[103]
Giant panda breast milk EVs	Immune system development and transfer of the genetic materials to the newborn cubs.	[104]
Zebrafish osteoblasts derived EVs	Involved in the maturation of osteoclasts.	[105]
Oviduct derived EVs	Improve the quality and development of the embryo.	[106]
	Transport neurohormones to the embryo, and regulate the reactive oxygen in vitro.	[107]
Plasma-derived EVs	Healing of cardiac cells.	[57]
Astrocyte derived EVs	Neurodegeneration function.	
Mesenchymal stromal cells derived EVs	Therapeutic function.	[108]
Umbilical cord-derived EVs	Immunomodulatory function.	

## Data Availability

Not applicable.
